# Mineral and Bone Disease in Black African Hemodialysis Patients

**DOI:** 10.5812/numonthly.9509

**Published:** 2013-08-13

**Authors:** Jaume Almirall

**Affiliations:** 1 Department of Nephrology, Departament de Medicina, Universitat Autónoma de Barcelona, Sabadell, Spain

**Keywords:** Renal Dialysis, Hyperparathyroidism

***Dear Editor,***

We read with interest the article by Seck SM et al. (1) recently has appeared in the Journal, in which they describe the epidemiological patterns of CKD-MBD in end-stage renal disease patients undergoing chronic hemodialysis in Senegal.

First we would like to underline the interest of such studies made in countries with a particular situation with limited resources, where dialysis access is still poor and complementary therapies as phosphate binders, vitamin D analogs or Cinacalcet are restricted or prohibitive. All of these conditions make difficult to compare their results with those obtained in Western countries. Nevertheless, the reported results are not bad, with 32%, 39% and 35% of patients in the recommended guidelines for calcium, phosphate and parathyroid hormone (PTH). 

In any case, there are some aspects that we would like to comment. As the authors mention, interpretation of parathormone levels in black subjects might be cautious. It is well known that black patients have higher parathyroid gland mass and circulating PTH levels than whites. This may predispose black patients to more severe parathyroid disease when renal failure develops (2). Nevertheless, how the correlations between PTH levels and bone turnover apply to black patients is less known, since black patients may have a relative resistance to PTH.

A major determinant of hyperparathyroidism development, other than race, is serum phosphorus (3), in this line we want to add some comments. As the authors explain, all patients were dialysed with a 1.75 mmol/L calcium dialysate, the question is why? KDIGO CKD–MBD recommendation’s state in point 4.1.3 to use a dialysate calcium concentration between 1.25 and 1.50 mmol/L (2.5 and 3.0 mEq/L) (2D) (4). I believe that practice could have some favorable consequences: for patients with a dynamic bone disease, for better control of valvular and peripheral vascular calcifications. Also, considering the importance of control of phosphorous, using les calcium in dialysate can allow a more liberal use of calcium salts that are inexpensive. Obviously, 1.75 mmol/L calcium dialysate could be indicated in some particular patients, but not in a generalized form. 

The last comment is on 25(OH) vitamin D levels. In "Patients and Methods" the authors report they measured calcidiol every six months, but nothing is referred in the results or in the logistic regression analysis. This could be an interesting point to add for further investigation in order to explain secondary hyperparathyroidism.
